# Assessing an active distracting technique during primary mandibular molar pulpotomy (randomized controlled trial)

**DOI:** 10.1002/cre2.702

**Published:** 2022-12-08

**Authors:** Ekram Alsibai, Nada Bshara, Hasan Alzoubi, Laith Alsabek

**Affiliations:** ^1^ Department of Pediatric Dentistry, Faculty of Dentistry Damascus University Damascus Syria

**Keywords:** child, dental anxiety, video games

## Abstract

**Objectives:**

This study aims to evaluate the effectiveness of two different distraction techniques (Audio Video Distraction/Video Game Distraction) in the management of anxious pediatric patients during dental treatment.

**Materials and Methods:**

One hundred and five children were randomly divided into three groups; Group A: active distraction using video games on a tablet device and wireless joystick (VG). Group B: passive distraction using video on tablet, and wireless headphones (AV). Group C (Control group): basic behavior guidance technique Tell Show Do was used (C). The children were selected from the department of pediatric dentistry at the Faculty of Dentistry, Damascus University, who required pulpotomy in primary mandibular molars. All children were assessed by: Simplified Wong‐Baker FACES for pain scale (self‐report), and “HOUPT” Behavior Rating Scale for Overall Behavior (non‐self‐report), at the end of treatment.

**Results:**

One hundred and five children completed the study (57 boys and 48 girls) aged between 6 and 10 years (mean age of 7.4 years). The active distraction (VG) group was superior to the passive distraction (AV) group and the control group (C) on the pain scale with statistically significant differences as appeared in Simplified Wong‐Baker Scale (*p* = .000), The active distraction (VG) group was superior to the control group (C) in overall behavior as appeared in HOUPT scale (*p* = .041), but it was no statistically significant differences between (VG) group and (AV) group in overall behavior (*p* = .605). With the use of Bluetooth technology and wireless devices, the workspace was comfortable for the dentist and did not interfere with the movement of his hands.

**Conclusion:**

Positive distraction with video games by wireless joystick displayed on the portable tablet on the dental chair was the best technique for reducing dental anxiety and reported pain in school children (6‐10 years) and was better than negative distraction by video cartoons on the tablet device.

## 1 INTRODUCTION

The foundation of pediatric dental practice is the ability to establish a good dental experience (Avery's et al., 2021). Dental anxiety is common 6%–20% among children aged 4–18 years (Klingberg & Broberg, 2007). This rate increased in 2019 to reach up to 42% in different populations (Prado et al., 2019). Dealing with uncooperative patients is one of the most important challenges for dentists (York et al., 2007).

For many years the process of leading a child in the dental clinic was called “behavior management” but in 2005 the term “Behavior Guidance” was adopted in the recommendations of the American Academy of Pediatric Dentistry (AAPD) to emphasize that the goals of behavior guidance are not to “deal with” the child's behavior but to establish communication with the child and with his parents to obtain good oral health (Avery's et al., 2021).

Distraction is a technique that dissipates the child from the painful stimulus and allows for successful and high‐quality treatment (Al‐Khotani et al., 2016). Distraction is divided into two types: Passive Distraction which let the child remain calm while the dentist is doing a distraction such as watching videos, listening to music with headphones, reading a book to the child, or telling him a story (Srouji et al., 2010). Active (positive) distraction: It depends on encouraging the child to participate in certain activities during the procedures, and it includes singing songs, pressing a ball with the hand, taking a deep breath, and playing with electronic devices (Srouji et al., 2010).

The number of clinical studies on the effectiveness of the distraction technique in the management of dental fear and anxiety in children and adults during dental treatment is very low, and there is no consensus in methodologies and research results, which indicates the need for more controlled clinical studies on this technique (Prado et al., 2019).

Distraction is defined as a behavior management technique that relieves pain and behavioral disturbance by diverting children's attention away from painful stimuli during extensive dental procedures (Aminabadi et al., 2012). The distraction technique is a safe strategy that relies on the patient's limited attention span and diverts his attention from unpleasant actions (Flores et al., 2022).

The distraction can be negative or positive (active or passive), where audiovisual distraction is a type of negative distraction that affects two types of senses, hearing and vision, while playing video games is a positive distraction that affects an additional sense, which is the kinesthetic sense (Allani & Setty, 2016).

Active distraction depends on the child participating in certain activities during the procedures and includes singing songs, pressing a ball with the hand, taking deep breaths, and playing with electronic devices (Srouji et al., 2010). Many studies have done active distractions using children's toys such as coloring and wooden toys, making different movements of hands and feet, or drawing in the air to distract the child from painful treatments (Abdelmoniem & Mahmoud, 2016; Debs & Aboujaoude, 2017).

## 2 MATERIALS AND METHODS

### 2.1 Study design

This study was designed as a randomized controlled trial (RCT) to compare the effectiveness of (VG) distraction and (AV) distraction to a control group during pulpotomy in primary mandibular molars in 6–10‐year‐old children.

This clinical trial was conducted in the Department of Pediatric Dentistry at Damascus University in protocol record (IRB No. UDDS‐1987‐15082019/SRC‐1450) and approved by ClinicalTrials.gov (NCT05191836). After ethical consent was obtained, informed consent was obtained from parents or guardians to participate in this study.

### 2.2 Sample size calculation

The sample size was calculated using G‐Power 3.1 statistical program with (*α* = .05, and power = .95). The effect size was calculated according to previous studies (Mohammed et al., 2018). The sample size was 105 children, (35 children in each group) (Figure 2).

### 2.3 Patient selection

Children were selected from the Department of Pediatric Dentistry, Damascus University, Faculty of Dentistry, based on the following inclusion criteria:

1. Children required pulpotomy in a primary mandibular molar.
2. Aged between 6 and 10 years.
3. Children were categorized definitely positive or positive ratings on the Frankl scale.

Exclusion criteria:

1. Children with a previous dental experience.
2. Children with systemic or mental disorders.

### 2.4 Randomized allocation

The children were randomly assigned according to the website www.randomaization.com into three groups.

### 2.5 Intervention

**Figure 1:**
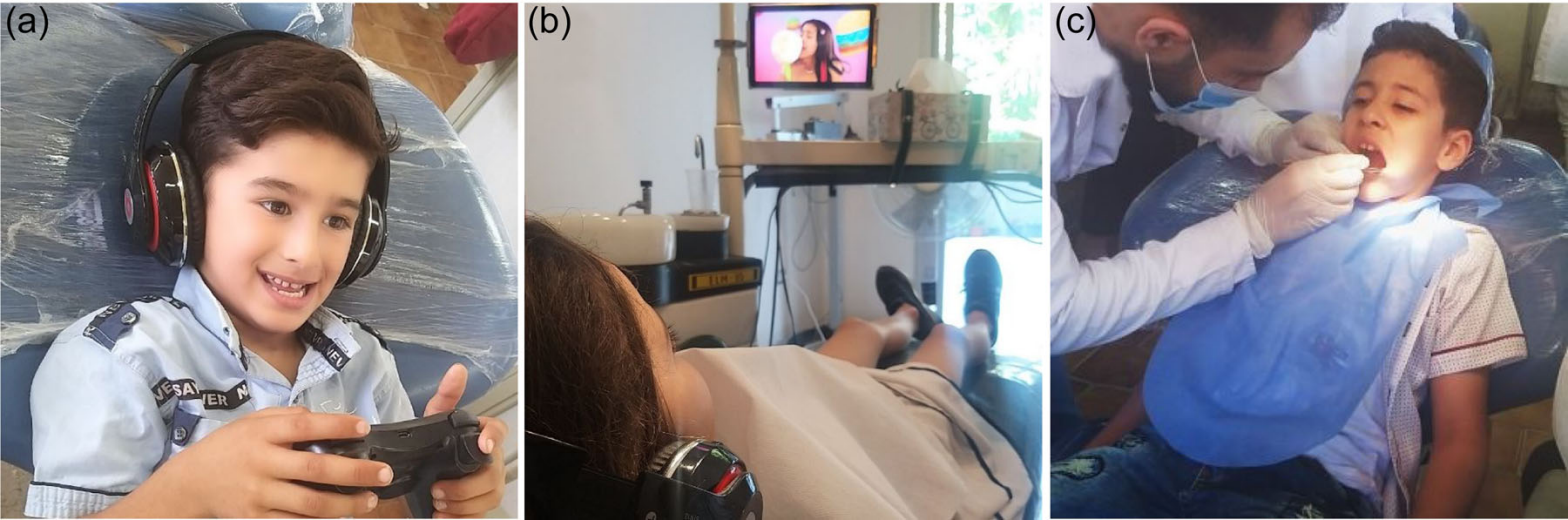
Different techniques used in the study: (a) active distraction, (b) passive distraction, and (c) Tell‐Show‐Do.

The pulpotomy technique was performed for all children following the standard protocol and the groups were divided according to the method of distraction (Figure 1):

Group A: (VG) active distraction with using video games on a tablet device, wireless joystick, and wireless headphones.

Group B: (AV) passive distraction with using video on tablet and wireless headphones.

Group C: (C) Control group with basic behavior guidance techniques (tell show do) and without using any type of distraction aids.

The child was interviewed, examined, and his degree of cooperation was evaluated according to the Frankl Behavior Rating Scale definitely positive or positive. After the child sat on the dental chair, he was chosen the electronic game for the (VG) group and the cartoon film for the (AV) group.

For group A (VG): The children were taught how to play through a wireless controller (joystick) and put on wireless headphones, then played for 5 min before starting the treatment and continued to play during the entire procedure.

For group B (AV): The child watched his favorite movie for 5 min before starting the treatment with headphones and continued watching during the entire procedure.

For group C (C): The traditional methods of managing behavior were followed without any intervention. The technique (Tell ‐ Show ‐ Do) was followed.

### 2.6 Pain assessment scale

The Simplified Wong‐Baker FACES pain rating scale was used in this study due to the reliability that was proved by Haji‐Bakr‐and AL‐monakel (Haji. Baker Rasha, 2015).

After completing the dental treatment and removing the rubber dam, the children were immediately asked to fill a face of the scale that described how they felt during the procedure while they was still on the dental chair in the treatment room (Figure 3).

### 2.7 Behavior assessment scale

The Behavior Rating Scale “HOUPT” for Overall Behavior. The external observer was selected 1 of the 6 scales at the end of treatment. Scales are shown in (Figure 4). The Simplified Wong‐Baker FACES and The Behavior Rating Scale “HOUPT” was measured after the final restoration and removal of the rubber dam.

**Figure 2:**
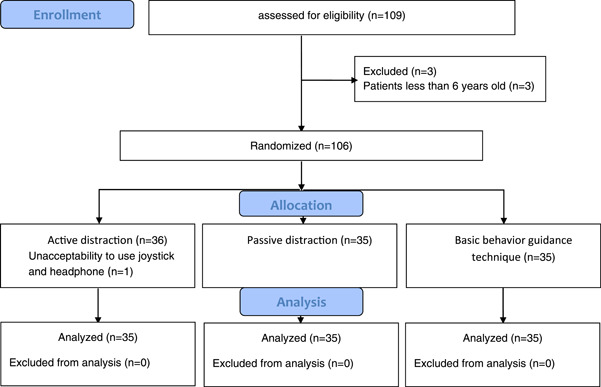
Consort flow diagram.

**Figure 3:**
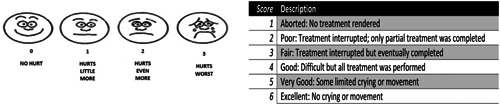
Simplified Wong–Baker FACES pain rating scale (Baker & Bashir al‐Mangal, 2015).

**Figure 4:**
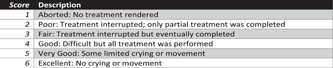
“HOUPT” Scale for overall behavior.

### 2.8 Statistical analysis

The statistical analysis was carried out using SPSS 24.0. One‐way ANOVA was used to study the differences between the three groups according to behavioral scale scores (HOUPT score, Simplified Wong‐Baker faces scale).

## 3 RESULTS

A total of 105 children, (57 boys and 48 girls, mean age 7.4) completed the study. In all groups, pain and behavioral scale scores (Simplified W‐B Faces, HOUPT) were recorded. One‐way ANOVA statistical test was done, and a significant difference was noticed between three groups on the overall behavior scale *p* = (.041) and a significant difference was noticed between three groups on the pain scale *p* = (.000) as shown in (Table 1). The Bonferroni test was applied for multiple‐dimensional comparisons to identify the trend of statistically significant differences.

**Table 1:** One‐way ANOVA to study the difference in three groups (*p* value < .05)

Scale	*p*‐value
HOUPT scale	.041
Simplified W‐P FACES	.000

There was a statistically significant difference in pain scale between Group A (VG) and Group B (AV) (*p* = .020) and between Group A (VG) and Group C (C) (*p* = .000) toward Group A, so the use of video games reduces the pain described by children. While there are no statistically significant differences between Group B (AV) and Group C (C) (*p* = .665), as shown in (Table 2).

**Table 2:** Bonferroni test for multiple correction comparison for pain reporting

Group	Source	Means difference	*p*‐value	Decision
Group A	Group B	−0.514	.020	Statistical differences Towards Group A
	Group C	−0.743	.000	Statistical differences Towards Group A
Group B	Group C	−0.229	.665	No statistical difference

There was a statistically significant difference in the overall behavior scale between Group A (VG) and Group C (C) (*p* = .035). On the other hand, there were no statistically significant differences compared to the overall behavior scale between the Group A(VG) and Group B (AV) (*p* = .605), as well as between Group B (AV) and Group C (C) (*p* = .605), as shown in (Table 3).

**Table 3:** Bonferroni test for multiple correction comparison for overall behavior (*p* value < .05)

Group	Source	Means difference	*p*‐value	Decision
Group A	Group B	0.343	.605	No statistical difference
	Group C	0.686	.035	Statistical differences Towards Group A
Group B	Group C	0.343	.605	No statistical difference

This indicates that the use of video games with joystick and portable tablet added a significant improvement in the behavior of children and reduce the self‐reported pain during the primary mandibular molar pulpotomy.

All connections were via Bluetooth and we did not use any wired devices so that the workspace remained empty and didn't interfere with the movement of the doctor's hands.

There were no statistically significant differences between males and females in three groups according to the scales used in the research HOUPT Scale and W‐B Scales, as shown in (Table 4).

**Table 4:** T‐test in Group(B)(V) multiple correction comparison for overall behavior (*p* value < .05)

Group	Scales	Gender	Mean	SD	*T*‐test	*p*‐Value	Result
Group A	**HOUPT Scale**	Male	5.63	0.576	1.182	.246	no statistical differences
		Female	5.36	0.674			
	**W‐B Scale**	Male	0.75	0.897	0.358	.722	no statistical differences
		Female	0.64	0.809			
Group B	**HOUPT Scale**	Male	4.59	1.098	1.291	.206	no statistical differences
		Female	5.08	1.038			
	**W‐B Scale**	Male	0.91	1.192	0.387	.701	no statistical differences
		Female	1.08	1.320			
Group C	**HOUPT Scale**	Male	5.67	0.488	1.244	.223	no statistical differences
		Female	5.35	0.862			
	**W‐B Scale**	Male	0.60	0.910	0.806	.427	no statistical differences
		Female	0.88	1.054			

## 4 DISCUSSION

Dental fear and anxiety have an impact on the quality of dental treatment (Milgrom et al., 2010). Dental treatment usually includes violent procedures, repeated injections, and the use of sharp tools at high speed, and may extend over several visits. These aspects affect the child's ability to tolerate dental treatment, which poses a challenge to the dentist, and therefore it is impossible to perform a successful dental treatment if the child's behavior is not controlled (Anthonappa et al., 2017).

Therefore, many procedural, behavioral, and nonpharmacological techniques have been proposed to reduce pain and discomfort during pediatric dental treatment. This study was conducted to compare different distraction techniques (active distraction–passive distraction) in reducing perceived pain during pulpotomy in children.

There are many scales for classifying behavior in children, the most famous of which is the Frankel Scale, which divides behavior into four categories (Definitely negative, Negative, Positive, and Definitely positive) (Dean, 2021). In this study, only children with positive or definitely positive behavior were included. This study was conducted to compare different distraction techniques (active distraction–passive distraction) in relieving pain during pulpotomy on primary mandibular molar in children aged 6–10 years old.

This study dealt with a new technique that a child plays video games through a wireless joystick linked via Bluetooth with a portable tablet on the dental chair and placing Bluetooth headphones during pulpotomy, starting from IAN injection (Inferior Alveolar Nerve Block) and ending with the final restoration, because of the use of Bluetooth Dental treatment usually includes painful procedures, repetitive injections, use of sharp tools at high speed, and frequent visits. These aspects affect the child's ability to tolerate treatment, which poses a challenge to the dentist and, therefore, it is difficult to achieve successful dental treatment if the child's behavior is not controlled technology and wireless devices, the workspace was comfortable for the dentist and did not interfere with the movement of his hands, The tablet was placed in a high position on the dental chair, so the child raised his head high, which facilitated treatment.

As our study results show, the use of the joystick and video games added a significant improvement in the behavior of children and the self‐reported pain during the dental treatment procedure, so that all children in active distraction group were treated with acceptable and satisfaction general behavior according to the scales used in the research. Thus, our method of distraction outperformed the passive distraction using video films. this is due to the physical activity of the child and they were blocked out from the surrounding environment, and our results agreed with many research that have been studied the effectiveness of active distraction through video games on a tablet device while performing different dental procedures, and their results showed that active distraction is superior to passive distraction (Attar & Baghdadi, 2015; Pande et al., 2020).

In addition, active distraction using the joystick, which was fixed to the dental chair, was superior in pain control during pulpotomy compared to using AV distraction and control group, but it was similar in child behavioral management during pulpotomy compared to using AV distraction.

Active distraction using video games is superior to passive distraction using video games for pain described by children, although other studies used an effective technique in improving self‐reported pain, PlayStation video games did not affect overall behavior (Guinot et al., 2021). This is may due to blocking out the sounds of dental by the use of headphones in two techniques active and passive distraction.

Some studies have found that playing video games has improved a child's adaptation to the dentist, compared to watching cartoons while injecting local anesthesia. In addition, playing a video game and watching cartoons film during dental treatment reduced heart rate, compared to traditional distraction techniques (Kumprasert et al., 2021) As found in our study that the use of video games is the most effective distraction technique for reducing disruptive behavior while performing dental treatments.

On the other hand, some studies used effective distraction in reducing the vomiting reflex while taking the upper and lower alginate impression, and they used the Intellectual Colored Game, which distracted the child's attention during the stressful alginate impression (Kulkarni et al., 2021). This is consistent with our study, which demonstrated the effectiveness of active distraction in diverting the child's attention away from the disturbing cause.

In contrast to our study, some studies found that distraction did not provide additional advantages in reducing fear and pain when compared to traditional methods such as directing behavior and positive reinforcement, but it is a way to attract the child's attention and activate the child's nervous and emotional centers, which leads to relaxation (Al‐Khotani et al., 2016; Shekhar et al., 2022).

This study found that playing a video game during dental treatment could better method a child's cooperation with the dentist, compared with watching cartoons and other conventional distractions (Cozzi et al., 2021).

## 5 CONCLUSION

Within the limits of this study, it can be concluded that the use of video games via joystick on tablets and headphones gave the best result in relieving dental anxiety and pain during pulpotomy in children.

Although the use of cartoon films through the tablet and headphones did not reduce the described pain in children, it was acceptable in managing children's behavior when performing the dental treatment and may lead to desirable behaviors in future visits.

## DETERMINANTS

A limitation of this study was the inability to blind the external investigator from the use of joystick and headphones, and the unacceptability of some children to use joystick and headphones.

## AUTHOR CONTRIBUTIONS

Ekram AlSibai conceived the idea and provided the treatment. Ekram AlSibai contributed to the writing. Hasan Alzoubi to the documenting. Nada Bshara conceived the idea and supervised the treatment. Laith Al Sabek contributed to study design.

## CONFLICT OF INTEREST

The authors declare no conflict of interest.

## Data Availability

Data are available on request due to privacy from the corresponding author.

## References

[cre2702-bib-0001] Abdelmoniem, S. A. , & Mahmoud, S. A. (2016). Comparative evaluation of passive, active, and passive‐active distraction techniques on pain perception during local anesthesia administration in children. Journal of Advanced Research, 7, 551–556. 10.1016/j.jare.2015.10.001 27222759 PMC4856782

[cre2702-bib-0002] Al‐Khotani, A. , Bello, L. A. , & Christidis, N. (2016). Effects of audiovisual distraction on children's behaviour during dental treatment: A randomized controlled clinical trial. Acta Odontologica Scandinavica, 74, 494–501. 10.1080/00016357.2016.1206211 27409593 PMC4960510

[cre2702-bib-0003] Allani, D. S. , & V Setty, D. J. (2016). Effectiveness of distraction techniques in the management of anxious children in the dental operatory. IOSR Journal of Dental and Medical Sciences, 15, 69–73.

[cre2702-bib-0004] Aminabadi, N. A. , Erfanparast, L. , Sohrabi, A. , Oskouei, S. G. , & Naghili, A. (2012). The impact of virtual reality distraction on pain and anxiety during dental treatment in 4‐6 year‐old children: A randomized controlled clinical trial. Journal of Dental Research, Dental Clinics, Dental Prospects, 6, 117. 10.5681/2Fjoddd.2012.025 23277857 PMC3529924

[cre2702-bib-0005] Anthonappa, R. P. , Ashley, P. F. , Bonetti, D. L. , Lombardo, G. , & Riley, P. (2017). Non‐pharmacological interventions for managing dental anxiety in children. Cochrane Database of Systematic Reviews, 2017(6):CD012676. 10.1002/2F14651858.CD012676

[cre2702-bib-0006] Attar, R. H. , & Baghdadi, Z. D. (2015). Comparative efficacy of active and passive distraction during restorative treatment in children using an iPad versus audiovisual eyeglasses: A randomised controlled trial. European Archives of Paediatric Dentistry, 16, 1–8. 10.1007/s40368-014-0136-x 25416522

[cre2702-bib-0007] Avery's, M. A. , Jeffrey, A. , & Dean, D. (2021). Dentistry for the child and adolescent. Elsevier.

[cre2702-bib-0008] Cozzi, G. , Crevatin, F. , Dri, V. , Bertossa, G. , Rizzitelli, P. , Matassi, D. , Minute, M. , Ronfani, L. , & Barbi, E. (2021). Distraction using buzzy or handheld computers during venipuncture. Pediatric Emergency Care, 37, 512. 10.1097/PEC.0000000000001689 30601349

[cre2702-bib-0009] Dean, J. A. (2021). McDonald and Avery's dentistry for the child and adolescent‐E‐book. Elsevier Health Sciences.

[cre2702-bib-0010] Debs, N. N. , & Aboujaoude, S. (2017). Effectiveness of intellectual distraction on gagging and anxiety management in children: A prospective clinical study. Journal of International Society of Preventive & Community Dentistry, 7, 315–320. 10.4103/2Fjispcd.JISPCD_397_17 29387614 PMC5774051

[cre2702-bib-0011] Flores, A. M. A. , Gómez, M. R. , González, G. I. M. , Delgadillo, R. H. , Enriquez, S. N. , Cepeda, M. A. A. N. , Ruiz, A. D. L. S. , & Soto, J. M. S. (2022). Distraction techniques in children with dental fear and anxiety. International Journal of Applied Dental Sciences, 8, 513–516. 10.22271/oral.2022.v8.i1h.1469

[cre2702-bib-0012] Guinot, F. , Mercadé, M. , Oprysnyk, L. , Veloso, A. , & Boj, J. R. (2021). Comparison of active versus passive audiovisual distraction tools on children's behaviour, anxiety and pain in paediatric dentistry: A randomised crossover clinical trial. European Journal of Paediatric Dentistry, 22, 230–236. 10.23804/ejpd.2021.22.03.10 34544253

[cre2702-bib-0013] Baker, R. al‐H. , & Bashir al‐Manqal, M. (2015). A comparison study of preemptive paracetamol and ibuprofen oral administration to reduce the pain during and after primary molar extraction. University of Damascus.

[cre2702-bib-0014] Klingberg, G. , & Broberg, A. G. (2007). Dental fear/anxiety and dental behaviour management problems in children and adolescents: A review of prevalence and concomitant psychological factors. International Journal of Paediatric Dentistry, 17, 391–406. 10.1111/j.1365-263X.2007.00872.x 17935593

[cre2702-bib-0015] Kulkarni, P. , Chhattani, B. , Agrawal, N. , Mali, S. , Kale, S. , & Thakur, N. S. (2021). Management of gagging and anxiety in children by play way method. International Journal of Drug Research And Dental Science, 3, 35–40. 10.36437/ijdrd.2021.3.2.B

[cre2702-bib-0016] Kumprasert, P. , Prapansilp, W. , & Rirattanapong, P. (2021). Video games, audiovisual, and conventional distractions for pediatric dental patients: A crossover randomized controlled clinical trial. Mahidol Dental Journal, 41, 225–234.

[cre2702-bib-0017] Milgrom, P. , Newton, J. T. , Boyle, C. , Heaton, L. J. , & Donaldson, N. (2010). The effects of dental anxiety and irregular attendance on referral for dental treatment under sedation within The National Health Service in London. Community Dentistry and Oral Epidemiology, 38, 453–459. 10.1111/j.1600-0528.2010.00552.x 20545723 PMC2945617

[cre2702-bib-0018] Mohammed, A. H. , Nada, B. , & Zuhair, A. N. (2018). Effectiveness of audio visual distraction using virtual reality eyeglasses versus tablet device in child behavioral management during inferior alveolar nerve block. Anaesthsia Pain Intensive Care, 22(1), 55–61.

[cre2702-bib-0019] Pande, P. , Rana, V. , Srivastava, N. , & Kaushik, N. (2020). Effectiveness of different behavior guidance techniques in managing children with negative behavior in a dental setting: A randomized control study. Journal of the Indian Society of Pedodontics and Preventive Dentistry, 38, 259–265. 10.4103/JISPPD.JISPPD_342_20 33004724

[cre2702-bib-0020] Prado, I. M. , Carcavalli, L. , Abreu, L. G. , Serra‐Negra, J. M. , Paiva, S. M. , & Martins, C. C. (2019). Use of distraction techniques for the management of anxiety and fear in paediatric dental practice: A systematic review of randomized controlled trials. International Journal of Paediatric Dentistry, 29, 650–668. 10.1111/ipd.12499 30908775

[cre2702-bib-0021] Shekhar, S. , Suprabha, B. S. , Shenoy, R. , Rao, A. , & Rao, A. (2022). Effect of active and passive distraction techniques while administering local anaesthesia on the dental anxiety, behaviour and pain levels of children: A randomised controlled trial. European Archives of Paediatric Dentistry, 23, 417–427. 10.1007/s40368-022-00698-7 35274286 PMC9167192

[cre2702-bib-0022] Srouji, R. , Ratnapalan, S. , & Schneeweiss, S. (2010). Pain in children: assessment and nonpharmacological management. International Journal of Pediatrics, 2010, 2010. 10.1155/2010/474838 PMC2913812 20706640

[cre2702-bib-0023] York, K. M. , Mlinac, M. E. , Deibler, M. W. , Creed, T. A. , & Ganem, I. (2007). Pediatric behavior management techniques: A survey of predoctoral dental students. Journal of Dental Education, 71, 532–539. 10.1002/j.0022-0337.2007.71.4.tb04306.x 17468315

